# Design and Applications of Extracellular Matrix Scaffolds in Tissue Engineering and Regeneration

**DOI:** 10.3390/cells14141076

**Published:** 2025-07-15

**Authors:** Sylvia Mangani, Marios Vetoulas, Katerina Mineschou, Konstantinos Spanopoulos, Maria dM. Vivanco, Zoi Piperigkou, Nikos K. Karamanos

**Affiliations:** 1 Biochemistry, Biochemical Analysis & Matrix Pathobiology Research Group, Laboratory of Biochemistry, Department of Chemistry, University of Patras, 26504 Patras, Greece; sylvia.mangani@upatras.gr (S.M.); up1087042@ac.upatras.gr (M.V.); up1086993@ac.upatras.gr (K.M.); up1087032@upatras.gr (K.S.); zoipip@upatras.gr (Z.P.); 2 CIC bioGUNE, Basque Research and Technology Alliance, BRTA, Technological Park of Bizkaia, 48160 Derio, Spain; mdmvivanco@cicbiogune.es

**Keywords:** extracellular matrix scaffolds, tissue engineering, decellularized scaffolds, multidimensional bioprinting

## Abstract

Tissue engineering is a growing field with multidisciplinary players in cell biology, engineering, and medicine, aiming to maintain, restore, or enhance functions of tissues and organs. The extracellular matrix (ECM) plays fundamental roles in tissue development, maintenance, and repair, providing not only structural support, but also critical biochemical and biomechanical cues that regulate cell behavior and signaling. Although its specific composition varies across different tissue types and developmental stages, matrix molecules influence various cell functional properties in every tissue. Given the importance of ECM in morphogenesis, tissue homeostasis, and regeneration, ECM-based bioscaffolds, developed through tissue engineering approaches, have emerged as pivotal tools for recreating the native cellular microenvironment. The aim of this study is to present the main categories of these scaffolds (i.e., natural, synthetic, and hybrid), major fabrication techniques (i.e., tissue decellularization and multidimensional bioprinting), while highlighting the advantages and disadvantages of each category, focusing on biological activity and mechanical performance. Scaffold properties, such as mechanical strength, elasticity, biocompatibility, and biodegradability are essential to their function and integration into host tissues. Applications of ECM-based bioscaffolds span a range of engineering and regenerative strategies, including cartilage, bone, cardiac tissue engineering, and skin wound healing. Despite promising advances, challenges remain in standardization, scalability, and immune response modulation, with future directions directed towards improving ECM-mimetic platforms.

## 1. Introduction

Tissue engineering is a fast-advancing, interdisciplinary field focusing on biological tissue restoration or replacement, through the interdisciplinary application of principles and techniques from biology, materials science, and engineering [1]. A central objective in this field is the development of functional tissue constructs by integrating multiple cell types, biomaterials, and signaling molecules [2]. The extracellular matrix (ECM) serves as the crucial, non-cellular macromolecular three-dimensional (3D) meshwork of macromolecules, with a particular role in guiding tissue development and homeostasis [3,4]. ECMs are mainly composed of water, (glyco)proteins, proteoglycans (PGs), and heteropolysaccharides, such as hyaluronan; however, each tissue type establishes a unique matrix composition and architecture to maintain its structural integrity and, by extension, that of the associated organ [5,6].

Although once considered a passive bystander in cellular processes, ECM acts as a silent architect of cellular behavior, from the earliest stages of embryogenesis through adulthood, modulating tissue development and homeostasis [7]. Beyond providing structural support to the cells within the tissues, matrix macromolecules generate biochemical/biomechanical cues that mediate cell morphology, mechanosensing, signaling, spatial organization, and intercellular communication [8]. The main ECM components include collagens, elastin, laminin, fibronectin, PGs, and glycosaminoglycans (GAGs) [9]. Furthermore, ECM is a dynamic reservoir for various growth factors, including fibroblast growth factor (FGF), epidermal growth factor (EGF), insulin-like growth factor 1 (IGF-I), transforming growth factor (TGF-α, -β), vascular endothelial growth factor (VEGF), platelet-derived growth factor (PDGF), keratinocyte growth factor, hepatocyte growth factor, neural growth factor (NGF), and bone morphogenic proteins (BMPs). These ECM-sequestered factors are released in a tightly regulated manner, guiding stem cell differentiation and further tissue development, such as angiogenesis (i.e., VEGF, FGF, PDGF, and TGF-β), cartilage formation (i.e., BMPs, TGF-β, IGF-I, FGF, PDGF), bone formation (i.e., BMP2, BMP4, TGF-β, FGF), wound healing (i.e., TGF-β, EGF, FGF, VEGF, PDGF), neurogenesis (i.e., NGF, FGF-2, IGF-I, BDNF, VEGF), and cardiac development (i.e., TGF-β, FGF-9, IGF-2), among others [10,11,12,13].

ECM stiffness is pivotal in mechanotransduction mechanisms, affecting cell fate responses and lineage specification [14]. For instance, soft matrices promote neuron differentiation, while stiffer matrices favor osteogenesis [15]. Furthermore, collagen crosslinking, often mediated by lysyl oxidases (LOX) and LOX-like proteins, leads to excessive ECM accumulation that triggers tissue scarring and disrupts organ functionality during fibrotic conditions [16]. Studies have shown that LOX inhibition can reduce tissue fibrosis, resulting in lower tumor metastatic potential, exhibiting a crucial link between cancer and fibrosis [17].

Given the predominant role of ECM macromolecules in both tissue development and various pathological conditions, the synthesis of ECM-based platforms is of critical importance for advancing tissue engineering applications [18]. This study presents and critically analyzes the techniques utilized in the design of ECM-inspired models and their applications in tissue regeneration and engineering approaches.

## 2. ECM-Based/Mimetic Bioscaffolds Utilized in Tissue Engineering-Design Strategies

In tissue engineering, ECM-derived biomaterials act as nature’s architectural blueprints, closely mimicking the complex environments where cells adhere, grow, and differentiate. To this regard, they exhibit multifunctional roles—offering structural support while also providing mechanical and biochemical cues that guide and coordinate tissue regeneration [19,20]. ECM-based platforms utilized in tissue engineering can be classified into three main categories, depending on the source of the utilized monomers; natural, synthetic, and hybrid [21]. Natural scaffolds are typically derived from biological sources and closely replicate the composition of native ECMs, hence preserving the structural integrity and biochemical cues essential for mediating cellular functions [22]. Conversely, synthetic scaffolds composed of artificially synthesized (lab-engineered) polymers, enable precise control of mechanical properties, including strength, stiffness, elasticity, and porosity. Finally, hybrid composites are designed to integrate both natural ECM components alongside with synthetic materials, merging the bioactivity of biological components with the mechanical strength of synthetic ones, thereby offering a promising remedy for various tissue engineering and regenerative medicine applications [23].

Scaffold fabrication techniques are tailored to address specific mechanical, physicochemical, and biological properties, depending on the requirements of the impaired tissue or organ [24,25]. A summary of the diverse methodologies utilized in the design of bioscaffolds applied in tissue engineering and biomedical applications are given in Table 1. Among them, tissue/organ decellularization and multidimensional bioprinting are further discussed in detail.

### 2.1. Decellularized ECM Scaffolds

Developing bioscaffolds that closely mimic native tissue structure while also minimizing host immune rejection is a significant challenge in tissue engineering as adverse immune responses are possible. Decellularized ECM (dECM) scaffolds offer a promising platform for tissue engineering and regeneration, emerging as a key approach to eliminate immune-related complications [34,35]. Decellularization is the process of removing cells and their components while preserving the native ECM structural and functional microenvironmental characteristics, thereby serving as a natural bioscaffold for engineering/regenerative strategies [36]. Notably, the removal of cellular components and antigens from tissues is essential to reduce immune and inflammatory responses, while the preservation of matrix structural macromolecules and growth factors mediates cell functional properties, including adhesion, proliferation, differentiation, and migration [37,38,39].

**Figure 1:**
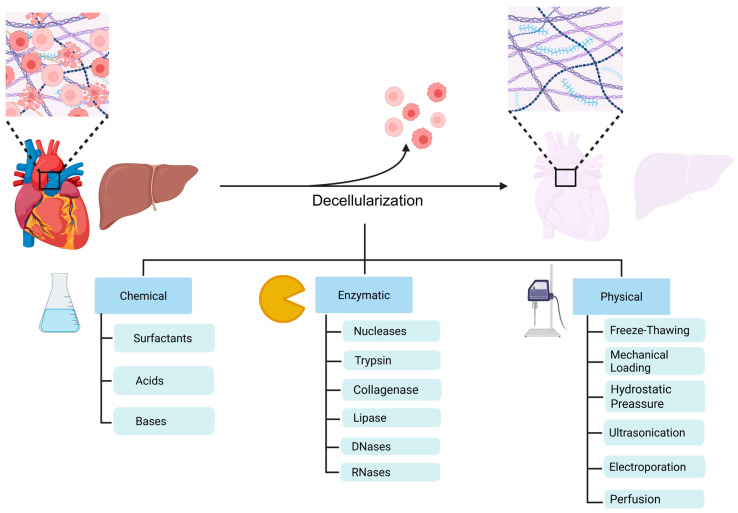
Major tissue/organ decellularization techniques for bioscaffolds fabrication. Decellularization approaches designed to eliminate cellular components and antigens, while maintaining the structural integrity and functional properties of the native ECM. Decellularization methods are categorized into chemical, enzymatic, and physical categories, and may be used individually or in combination to enhance ECM preservation. Created with BioRender.com.

Decellularization can be achieved through various methods, which can be applied individually or in combination to optimize the removal of the cellular material. These techniques can be classified into three main categories (Figure 1); chemical, enzymatic, and physical, each of one presenting their unique advantages and disadvantages [40,41]. Perfusion-based techniques have been widely used for whole-organ decellularization, enabling the generation of bioartificial constructs for complex organs such as the heart, lung, kidney, and liver [42].

Surfactants are primarily used as chemical decellularization agents, and they are classified—depending on their charge—as ionic, non-ionic, or zwitterionic [42]. Particularly, ionic surfactants are able to solubilize the lipids (cell membrane disruption) and the intracellular cytoplasmic components, while also disrupting the DNA [42,43]. Sodium deoxycholate, sodium dodecyl sulfate (SDS), and Triton X-200 are the main representatives of this group [44]. Despite the elimination of a high percentage of nucleic acids, ionic surfactants have been shown to disrupt the ECM structure and the properties of various matrix components (impaired collagen integrity, reduced GAG content), as well as the availability of ECM-sequestered growth factors [43,44]. On the other hand, non-ionic surfactants, such as Triton X-100, can disrupt the cell membrane and DNA-protein interactions [27,36]. However, Triton X-100 exhibits less efficient cell lysis (e.g., in tendon decellularization), and its action appears highly tissue-dependent, meaning that it should be combined with other chemical techniques [44,45]. Zwitterionic detergents, such as 3-[(3-cholamidopropyl)-dimethylammonio]-1-propanesulfonate, have been shown to exhibit properties from both ionic and non-ionic detergents, thus better supporting cell removal from native tissues and more effectively preserving the ECM structure [46,47].

Acidic and alkaline solutions are also widely used in tissue decellularization as they induce the disruption of cell membrane and the consequent solubilization of cytoplasmic components and degradation of nucleic acids; however, regulation of the concentration and exposure time should be critically evaluated as the extreme pH conditions may cause tissue ECM degradation and structural disorganization [39,48]. Finally, hypotonic/hypertonic solutions and other chemical solvents are also utilized in tissue/organ decellularization [48,49].

Along with the aforementioned chemical methods, enzymatic decellularization is often employed. Nucleases (DNases and RNases), collagenases, trypsin, lipase, and dispase are enzymatic agents that remove any remaining cellular components and DNA, preserving the structural and biochemical matrix integrity (Figure 1) [50]. The most commonly used enzymatic agent is trypsin due to its specific proteolytic activity; however, overexposure of the tissue to trypsin can cause essential ECM components to degrade [36,51].

Physical decellularization methods consist of techniques that are often primarily used in conjunction with chemical and enzymatic techniques. The most commonly utilized methods include freeze-thawing, mechanical stress, hydrostatic pressure, ultrasonication, electroporation, and perfusion (Figure 1) [52]. During freeze–thaw cycles, cell lysis can be achieved by thermal shock and extreme low temperature alterations. However, this technique does not achieve complete tissue decellularization, as a substantial portion of DNA often remains unremoved [53]. High hydrostatic pressure is also effective at inducing cell lysis, although it does not adequately remove nucleic acids. To address this limitation, DNase is often employed to degrade and eliminate remaining DNA fragments [36,48]. Furthermore, mechanical loading techniques often utilize physical stress to induce cell lysis [36]. High-frequency ultrasonic waves are applied to promote tissue decellularization, often in combination with SDS, in order to improve the efficiency of the method and protect ECM proteins from damage [49]. Electroporation is another widely used physical technique for tissue decellularization that involves the application of electrical pulses to destabilize the integrity of cell membrane and create nanopores that lead to cell apoptosis [52]. Finally, perfusion involves the delivery of chemical agents to effectively remove cellular and nucleic acid material while preserving the structural integrity of the ECM [36].

### 2.2. Multidimensional Bioprinting: From 3D Era to 6D Bioscaffold Manufacturing

**Figure 2:**
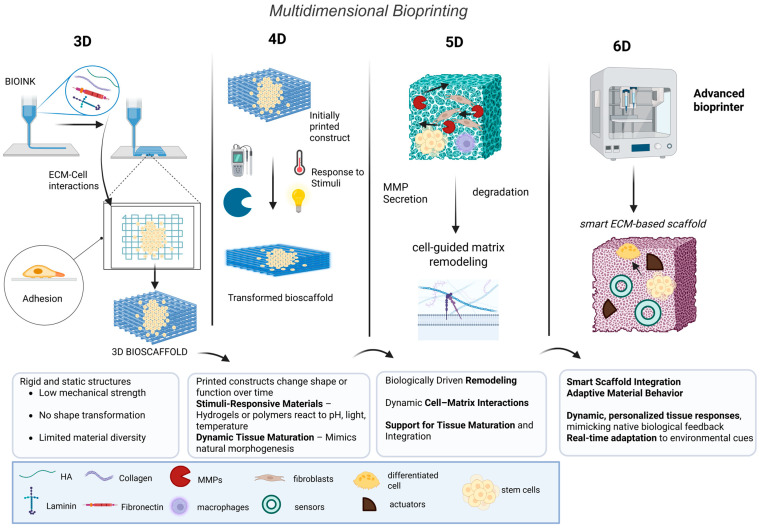
Schematic overview of multidimensional bioprinting approaches. Unlike traditional 3D bioprinting, 4D printed scaffolds respond to internal or external stimuli. 5D bioprinting incorporates tissue remodeling capabilities, while 6D systems integrate biosensors to monitor the local environment and activate responses such as growth factor release, stiffness modulation, or shape change. Created with BioRender.com.

Advances in bioprinting have significantly expanded the opportunities to replicate complex ECM microenvironments by enabling the spatially controlled deposition of cells, bioactive molecules, and biomaterials, thereby emulating the architectural and functional complexity of native tissues [54,55,56]. Particularly, bioinks derived from ECM components—such as collagen, fibronectin, laminin, elastin, and GAGs (i.e., hyaluronan and chondroitin sulfate)—offer an inherently bioactive microenvironment (Figure 2, Table 2) [57]. dECMs, previously analyzed in detail, have emerged as one of the most promising ECM-based bioinks due to their tissue specificity and ability to preserve native biochemical and biomechanical cues after removal of cellular components [58].

The physicochemical properties of bioinks are critical to the success of 3D bioprinting [59]. Bioinks should be printable, biocompatible, and possess mechanical properties suitable for the requirements of the target tissue. Notably, ECM-based hydrogels or hybrid hydrogels (matrix components in combination with synthetic polymers, such as polyethylene glycol, polycaprolactone, and polyurethane) provide tunable stiffness, degradation rates, and bioactivity (Figure 2) [60]. For example, collagen-based hydrogels are frequently combined with synthetic polymers to enhance mechanical robustness while retaining cell-friendly biochemical features. Such combinations demonstrate efficacy in a variety of applications, including osteochondral regeneration, where the mechanical integrity of the scaffold must be balanced with the osteogenic properties of the ECM [44,56].

While 3D bioprinting allows the fabrication of structurally complex tissues, its limitation lies in the static nature of the printed constructs. Native tissues are not static, but are highly dynamic, capable of responding to environmental mechanical and biochemical stimuli. To overcome this limitation, the concept of four-dimensional (4D) bioprinting incorporates time as the fourth dimension (Table 2) [61]. In 4D bioprinting, the printed structure is designed to undergo dynamic transformations after fabrication, through alterations in shape, mechanical properties, or biochemical activity in response to internal or external stimuli (Figure 2) [62]. This is typically achieved by stimuli-responsive smart materials, such as shape-memory polymers or hydrogels that respond to changes in temperature, pH, humidity, light, or enzymatic activity [63,64]. These materials enable the printed constructs to transform in a predictable manner, mimicking the morphogenetic processes observed during natural tissue development [65]. For instance, the use of temperature-responsive hydrogels allows the reversible swelling or deswelling in response to physiological temperatures [66]. A thermoresponsive ECM-based hydrogel can be programmed to contract, expand, or fold into complex geometries after implantation, enhancing integration to host tissue or enabling site-specific delivery of therapeutic agents [62]. Furthermore, light-sensitive materials offer spatial and temporal control over scaffold properties, such as stiffness and degradation, providing the ability to modulate the cellular microenvironment during tissue maturation [61]. This responsive behavior enhances the functional fidelity of engineered tissues, allowing for the design of constructs that actively participate in the regenerative process [17].

The progression to five-dimensional (5D) bioprinting further incorporates biological functionality into the dynamic framework. In this approach, constructs are not only responsive to stimuli but are also capable of undergoing biologically-mediated remodeling (Figure 2, Table 2) [67]. The inclusion of ECM molecules in bioinks is essential to this process, as it allows cells to dynamically interact with and modify their microenvironment [68]. For example, hydrogels with enzymatically cleavable crosslinkers permit matrix degradation in response to cell-secreted proteases (i.e., matrix metalloproteinases, MMPs) [69]. ECM remodeling supports cell migration, tissue integration, and vascular invasion, essential features for the maturation of complex tissues [70]. Additionally, ECM-rich materials support the deposition of new matrix components by embedded cells, gradually replacing the scaffold with tissue-specific extracellular matrix [71].

In the evolving landscape of bioprinting, the concept of “6D bioprinting” is proposed as a forward-looking paradigm that builds upon the foundational principles of 3D, 4D, and 5D bioprinting technologies. While 3D bioprinting focuses on spatial deposition of bioinks in three dimensions, 4D bioprinting introduces the element of time, enabling printed constructs to undergo dynamic changes in shape, function, or composition in response to environmental stimuli, often through the use of shape-memory materials or stimuli-responsive hydrogels [72]. In contrast, 5D bioprinting incorporates two additional rotational degrees of freedom to enable printing along complex, non-planar geometries and anatomically accurate curvatures using multi-axis printing platforms [73].

The proposed sixth dimension in “6D bioprinting” extends these capabilities by integrating adaptive, intelligent systems that enable real-time feedback, monitoring, and modulation of the bioprinted construct post-fabrication. This dimension emphasizes the convergence of biosensing, smart materials, and artificial intelligence (AI)-driven feedback loops that allow constructs to autonomously sense and respond to biochemical, mechanical, or electrical changes in their microenvironment. For example, recent studies have described hydrogel scaffolds embedded with nanosensors capable of monitoring cellular metabolism or stress signaling in situ and bioinks engineered to undergo controlled remodeling in response to pH, temperature, or enzymatic cues [74,75]. Furthermore, integration of soft electronics and wireless communication systems within tissue constructs may allow external modulation or diagnostic interfacing, facilitating precision biofabrication and long-term monitoring.

The concept of six-dimensional (6D) bioprinting introduces a further layer of complexity, by incorporating programmable and adaptive properties to the bioprinted constructs. Ultimately, 6D bioprinting leverages advanced smart materials that not only respond to stimuli, but can also adapt their behavior in real-time based on environmental feedback (Table 2) [74,75]. These constructs can be equipped with biosensors and actuators that monitor local conditions and trigger appropriate biological or mechanical responses, such as the controlled release of growth factors, modulation of scaffold stiffness, or shape transformation (Figure 2) [76]. This level of interactivity enables bioprinted tissues to mimic the dynamic reciprocity observed in native tissue environments, where cells and surrounding ECM engage in feedback loops that regulate development, healing, and homeostasis [77]. For example, a 6D bioprinted scaffold can contain mechanoresponsive polymers that stiffen in response to increased mechanical load, thereby directing stem cells toward osteogenic or myogenic differentiation [78]. The integration of real-time biosensing with smart ECM-mimetic materials enables the design of constructs that actively adapt to physiological cues, such as inflammation, ischemia, or mechanical strain [77,79]. Finally, the development of closed-loop bioprinted systems, capable of sensing, processing, and responding to their environment, represents a shift from static, passive implants to dynamic, adaptive bio-interfaces [80].

This sixth dimension represents a shift toward interactive and intelligent tissue systems, where printed constructs are no longer static or passively responsive, but actively engaged in sensing, adapting, and communicating—characteristics essential for next-generation personalized medicine, dynamic tissue regeneration, and real-time therapeutic interventions. By incorporating these emerging capabilities, 6D bioprinting aims to transform static biofabrication into a dynamic, self-regulating, and patient-specific technology ecosystem.

## 3. Applications of dECM and ECM-Bioprinted Scaffolds in Tissue Engineering Approaches

dECM bioscaffolds are commonly used in bone tissue engineering due to their ability to closely replicate the native tissue, as they provide both structural support and essential biochemical signals that guide cell morphology and behavior [81]. More specifically, demineralized decellularized human epiphysial bone scaffolds have demonstrated the ability to mediate cell adhesion, migration, and bone matrix production in vitro; induced mineralization has also been demonstrated [82]. Clinically, dECM bovine bone grafts have been successfully applied in the reconstruction of zygomatic bone defects, mandibular defect repair, and tibial defect treatment [81]. Moreover, dECM components can be incorporated into 3D printed hydrogels to enhance cell properties [26]. Notably, 3D and 4D printing technologies are already utilized for the fabrication of personalized and complex bone scaffolds with controlled porosity and mechanical properties [78,83]. In addition, bio-piezoelectric scaffolds replicate bone’s electromechanical characteristics, thus promoting osteogenic differentiation. Ultimately, 3D/4D printing is utilized to develop smart bioscaffolds that respond to external stimuli, promoting faster bone regeneration [84,85].

dECM bioscaffolds have also shown significant promise for a variety of applications in cartilage tissue engineering. These scaffolds can be implanted into knee joint cartilage defects to promote repair and integration within the surrounding tissue [86]. Additionally, human non-cellular cartilage matrix powders have been used as bioscaffolds for cartilage engineering, particularly in combination with synovium-derived stem cells, to enhance regenerative potential [87]. Processed dECM cartilage biomatrices have been shown to be effective in vitro, supporting cell adhesion and proliferation, but also the synthesis of new cartilage-specific ECM by chondrocytes and fibroblasts [88]. In parallel, 3D bioprinting has enabled distinct applications for cartilage regeneration. Direct inkjet bioprinting allows for the deposition of chondrocytes directly into the chondral defect [89]. Patient-specific cartilage constructs that demonstrate ECM deposition and drive cell proliferation have been further developed using photo-cross-linkable dECM-based bioinks containing rabbit auricular chondrocytes [90]. Moreover, fiber-reinforced cartilage dECM scaffolds have been produced by 3D printing to support the repair of articular cartilage defects in rabbits [91]. Finally, hybrid printing approaches, such as inkjet bioprinting of chondrocytes onto electrospun fibers, have yielded mechanically enhanced constructs that exhibit cartilage matrix formation both in vitro and in vivo [89].

Beyond bone and cartilage, dECM bioscaffolds are increasingly applied in skin tissue engineering, serving as dermal or skin substitutes in clinical settings [92]. Products, such as AlloDerm^®^ (LifeCell Corporation, AbbVie company, Texas, USA) and MatriDerm^®^, (MedSkin Solutions Dr. Suwelack AG, Billerbeck, Germany) are extensively utilized to address full-thickness and deep partial-thickness burn wounds, as well as to repair various soft tissue defects [93,94]. In parallel, marine-based biocomposites derived from fish or eel skin collagen blended with alginate have been employed as bioinks to create tissue engineering substitutes, demonstrating improved cell proliferation [95]. Complementing these, 3D bioprinting technologies enable the precise placement of both biomaterials and synthetic components, providing unique applications for skin regeneration [96]. Studies have shown the feasibility of in situ bioprinting of characteristic skin cells (i.e., fibroblasts and keratinocytes) directly onto wound sites, thus promoting rapid wound closure in mouse models [97]. Moreover, 3D bioprinting has been utilized to generate full-thickness engineered skin constructs encapsulating human primary skin cells [98]. All these pave the way for developing even more complex models, such as vascularized skin grafts utilizing multiple cell types and pigmented human skin constructs [96].

dECM nerve tissues serve as biological scaffolds for nerve regeneration by providing a 3D framework that supports axon attachment and directional growth toward target organs, and they have demonstrated promising results in in vivo animal models and in human clinical applications for neurological repair [99]. ECM-based hydrogels derived from sources such as the human umbilical cord, porcine bladder, or brain can support the differentiation of neural stem cells in vitro, thereby promoting axonal outgrowth [100]. Notably, implantation of brain-derived dECM in animal models has been shown to support neurological healing after traumatic brain injury [101]. Furthermore, decellularized meningeal scaffolds have been used to develop meningeal neuronal constructs with organized growth and intact axonal tracts, providing structural and biochemical cues for spinal cord injury regeneration [102]. Embedding decellularized peripheral nerve matrix within aligned collagen conduits significantly improved motor recovery and axonal alignment in rodent sciatic nerve models [103]. In addition to dECM-based methods, 3D bioprinting offers high precision in fabricating neural constructs, establishing it as a versatile tool for various neuroengineering approaches. Functional 3D neural mini-tissue constructs have been created by printing hydrogel bioinks encapsulating human neural stem cells [104]. Furthermore, scaffolds designed to promote axon regeneration and decrease glial scar deposition have been fabricated using 3D bioprinting for spinal cord injury repair in vivo [105]. Recently, graphene-infused neural dECM bioinks have been printed to form electrically conductive neural interfaces, enhancing neurite outgrowth and signal transmission [106]. This technology is also employed for creating disease models, such as glioblastoma, serving as platforms for high-throughput drug screening and assessing patient–specific treatment responses. Within the same framework, patient-specific nerve guide conduits can be created via bioprinting to facilitate peripheral nerve regeneration, potentially incorporating cells, composite hydrogels, or even growth factors [107].

Finally, dECM bioscaffolds offer a promising approach in tissue regenerative medicine for cardiac applications. Human cardiac matrices embedded with multiple cell types, including stem cells or cardiomyocytes, have been used as scaffolds for cardiac tissue regeneration [108]. dECM bioscaffolds can be also utilized for cell reseeding and subsequent engraftment into damaged myocardium as a cardiac bioprosthetic [109]. Seeding bioscaffolds with endothelial progenitor cells was shown to promote angiogenesis and reduce scar formation in infarcted rat hearts [110]. Also, injectable cardiac dECM microparticles have been demonstrated to preserve left ventricular function and improve ejection fraction in porcine myocardial infarction models [111]. In addition, 3D bioprinting techniques offer significant advantages in fabricating cardiac tissues with sophisticated architectures. Applications range from the development of microchannels to direct cardiomyocyte alignment and enhance beating strength, to the engineering of myocardial properties, such as anisotropy and vascularization, as well as the bioprinting of functional cardiac units for in vitro mechanistic studies and therapeutic screening [112]. Myocardium-on-chip models with dECM-tuned stiffness can be used to test drug cardiotoxicity [113]. Finally, hybrid bioprints combining cardiac dECM with conductive graphene oxide have been shown to enhance synchronous contraction and electrophysiological function in engineered heart tissues [114].

## 4. Conclusions and Perspectives

ECM-based bioscaffolds have become pivotal in tissue engineering as they provide a biologically active microenvironment that promotes cell adhesion, differentiation, and tissue remodeling. Tissue/organ decellularization, along with advancements in nanotechnology and multidimensional bioprinting, significantly enhance the mechanical and functional properties of the scaffolds. As research continues to merge biology with engineering, ECM-based scaffolds are set to play a key role in the future of tissue engineering and repair, with a focus on long-term clinical applications and regenerative medicine approaches. While still in its infancy, 6D bioprinting shows great potential for personalized regenerative medicine by enabling implants to adjust their behavior dynamically according to every individual’s case. Despite the widespread clinical adoption of ECM-based bioscaffolds over the past two decades, numerous practical limitations still hinder their broader clinical translation. Key challenges include the incomplete understanding of their mechanisms of action, difficulties in upscaling production, quality control, and integration into existing healthcare systems. Biologic variability, particularly due to animal-to-animal differences in ECM composition, contributes to a lack of chemical definition and reproducibility [115,116]. Additionally, concerns around immunogenic responses, the immunomodulatory effects of degradation products, and the absence of long-term or bioaccumulation studies limit confidence in their widespread use [117]. Structural constraints, such as the fixed geometry of ECM sheets, can further restrict their utility in minimally invasive procedure [118]. Moreover, unresolved issues in appropriate sterilization and disinfection methods, cell sourcing, regulatory classification, cost-efficiency, and the lack of robust preclinical data—especially in large animal models—pose further barriers [115,117,118,119,120]. Ethical considerations are also gaining prominence, particularly with personalized, matrix-based bioscaffolds. These include sourcing transparency, informed consent, and data handling [121]. As the field progresses, addressing these limitations through ethical frameworks, refined regulatory guidelines, and robust scientific validation will be essential for the successful implementation of ECM-based bioscaffolds in advanced tissue engineering and regenerative medicine approaches, as well as in personalized medicine applications.

## Figures and Tables

**Table 1:** Overview of key design techniques for bioscaffolds fabrication and their main applications in tissue engineering and regenerative medicine approaches.

Technique	ECM Involvement	Description	Applications	References
Decellularization	Direct ECM use	Removal of cells and nucleic acids from tissues; natural ECM mimic	Bone, gastrointestinal tract, respiratory system, vascular, and neural tissue engineering	[26,27]
Electrospinning	Mimic ECM	High voltage application; micro-/nano-structure fibers fabrication	Skin, bone, cartilage, heart, nerve repair and regeneration	[28]
Freeze-Drying Lyophilization	Mimic ECM	Porous scaffold fabrication; freeze-drying of polymer solution	Skin repair; bone, cardiac tissue and lung tissue engineering	[29]
Solvent Casting	No ECM	Porous scaffold fabrication; polymer blended with salt (porogen)	Bone engineering	[30]
Gas Foaming	No ECM	Porous scaffold fabrication; pores created by using high-pressure gas	Osteochondral regeneration	[31]
Multidimensional (3D/4D/5D/6D) Bioprinting	Use of ECM molecules as bio-ink	Layer-by-layer structures design; bioinks use (e.g., dECM-derived components or synthetic)	Skin, bone, muscle, cardiovascular system, respiratory system, digestive system engineering; neural and adipose tissue regeneration	[32]
Cryogel	Use of ECM molecules	Porous scaffold fabrication; freeze-thaw cycling	Bone and cartilage regeneration	[33]

Abbreviations: ECM, extracellular matrix; dECM, decellularized extracellular matrix; 3D, three-dimensional; 4D, four-dimensional; 5D, five-dimensional; 6D, six-dimensional.

**Table 2:** Comparison of multidimensional 3D, 4D, 5D, and 6D bioprinting for bioscaffold development. The references supporting the information in this table are cited in the corresponding sections of the main text.

Bioprinting Type	Main Bioink Component	Definition	Key Features	Advantages	Disadvantages
3D Bioprinting	Natural hydrogels: alginate, gelatin, collagen Synthetic hydrogels: PEG-based hydrogels ECM-derived bioinks: decellularized matrix	Layer-by-layer deposition of bioinks	Fixed 3D structure Compatible with various bioinks High spatial control	Simple and mature technology Precise shape control Cost-effective and scalable	No adaptability Limited ability to mimic dynamic biological functions
4D Bioprinting	Smart biopolymers like chitosan, PNIPAAm [poly(N-isopropylacrylamide)] Hydrogels with embedded nanoparticles for remote actuation	Scaffold changes shape and/or function over time in response to stimuli	Stimuli-responsive (i.e., pH, temperature) Dynamic transformation	Mimics tissue morphogenesis Enables smart drug delivery and/or shape change Improved biofunctionality	Complex material requirements Difficult to control transformations
5D Bioprinting	Reinforced bioinks: nanocellulose, nanoclay, graphene oxide for mechanical strength Hybrid hydrogels: GelMA with reinforcing fillers and nanoparticles for remote actuation	Additional rotational axes for more complex printing	Tissue-specific matrix scaffold High mechanical fidelity	Enhanced anatomical accuracy Ideal for curved or layered tissues	Mechanically complex High cost Motion challenges
6D Bioprinting	Hydrogels integrated with soft electronics (e.g., flexible conductive inks) Hybrid smart composites with AI-enabled interfaces or wireless modules	Fusion of 5D spatial flexibility and 4D dynamic responsiveness	Smart materials Multi-axis printing Dynamic and anatomical conformance	Ultimate precision and adaptability Mimic in vivo architecture Ideal for on-body printing	Extremely complex Research-use High cost and limited availability

## Data Availability

Not applicable.

## Abbreviations

The following abbreviations are used in this manuscript:

3D	Three-dimensional
4D	Four-dimensional
5D	Five-dimensional
6D	Six-dimensional
BMPs	Bone morphogenic proteins
dECM	Decellularized extracellular matrix
EGF	Epidermal growth factor
FGF	Fibroblast growth factor
IGF-I	Insulin-like growth factor 1
GAGs	Glycosaminoglycans
LOX	Lysyl oxidase
NGF	Neural growth factor
PDGF	Platelet-derived growth factor
PGs	Proteoglycans
SDS	Sodium dodecyl sulfate
TGF	Transforming growth factor
VEGF	Vascular endothelial growth factor
